# Pathological femoral fractures due to osteomalacia associated with adefovir dipivoxil treatment for hepatitis B: a case report

**DOI:** 10.1186/1746-1596-7-108

**Published:** 2012-08-20

**Authors:** Motoyuki Tanaka, Takao Setoguchi, Yasuhiro Ishidou, Yoshiya Arishima, Masataka Hirotsu, Yoshinobu Saitoh, Shunsuke Nakamura, Hironori Kakoi, Satoshi Nagano, Masahiro Yokouchi, Junichi Kamizono, Setsuro Komiya

**Affiliations:** 1 Department of Orthopaedic Surgery, Graduate School of Medical and Dental Sciences, Kagoshima University, Kagoshima, Japan; 2 The Near-Future Locomotor Organ Medicine Creation Course (Kusunoki Kai), Graduate School of Medical and Dental Sciences, Kagoshima University, 8-35-1 Sakuragaoka, Kagoshima, 890-8520, Japan; 3 Department of Medical Joint Materials, Graduate School of Medical and Dental Sciences, Kagoshima University, Kagoshima, Japan

**Keywords:** Osteomalacia, Pathological femoral neck fracture, Adefovir dipivoxil, Hepatitis B, Fanconi’s syndrome

## Abstract

We present a case of a 62-year-old man who underwent total hip arthroplasty for treatment of pathologic femoral neck fracture associated with adefovir dipivoxil-induced osteomalacia. He had a 13-month history of bone pain involving his shoulders, hips, and knee. He received adefovir dipivoxil for treatment of lamivudine-resistant hepatitis B virus infection for 5 years before the occurrence of femoral neck fracture. Orthopedic surgeons should be aware of osteomalacia and pathological hip fracture caused by drug-induced renal dysfunction, which results in Fanconi’s syndrome.

**Virtual slides:**

The virtual slide(s) for this article can be found here: http://www.diagnosticpathology.diagnomx.eu/vs/1600344696739249

## Background

Hypophosphatemic osteomalacia caused by proximal renal tubule dysfunction induces Fanconi’s syndrome, which leads to impaired reabsorption of amino acids, glucose, urate, and phosphate [1]. The chronic loss of phosphate and impaired synthesis of 1,25-dihydroxyvitamin D3 may lead to failure of bone mineralization. Recently, osteomalacia was reported in cases in which hepatitis B virus and human immunodeficiency virus (HIV) infections were treated using high-dose adefovir dipivoxil [2-6]. We report a case of a patient who underwent total hip arthroplasty for pathological femoral neck fracture associated with osteomalacia induced by low-dose adefovir dipivoxil treatment.

## Case presentation

**Figure 1:**
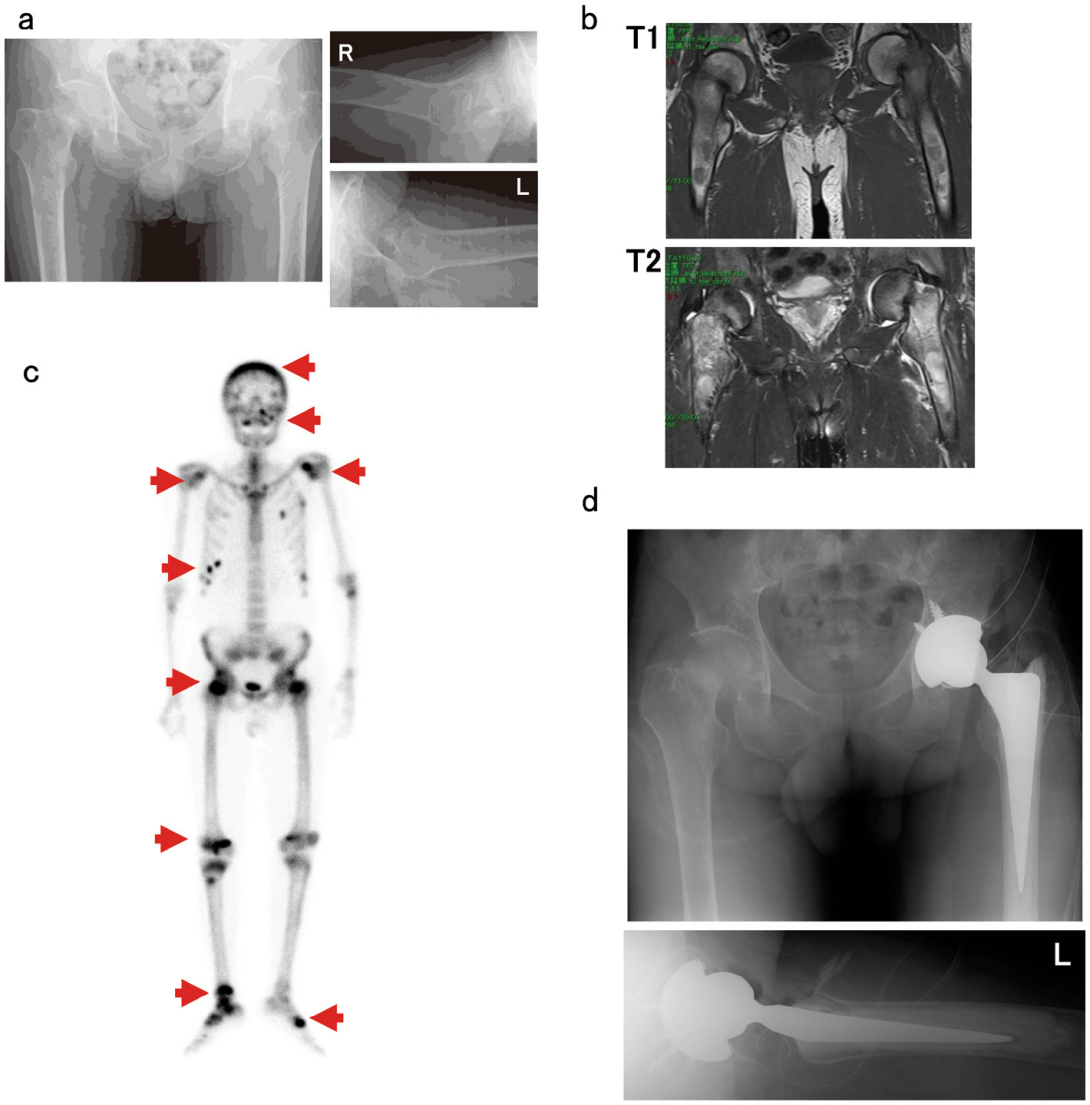
**Imaging studies.a**: Plain radiographs of both femurs reveal femoral neck fractures. (right, Garden III fracture; left, Garden IV fracture) **b**: Coronal T1-weighted image demonstrates low-intensity femoral neck fractures and the T2-weighted image shows high-intensity bone edema. **c**: ^99m^Tc-hydroxymethylene diphosphonate (HMDP) scintigraphy demonstrates significant abnormal uptake in calvaria, maxilla, both scapulae, ribs, both femoral necks, right condyle of the femur, right tibia, and both tarsi. **d**: Plain radiographs of hip joints in which total hip arthroplasty was performed by inserting an implant in the left hip joint.

A 62-year-old man started experiencing pain in the right knee and left shoulder pain in January 2010. He visited a clinic and was administered salazosulfapyridine and methylprednisolone therapy for rheumatoid arthritis. However, the pain gradually increased, and he started experiencing pain in his hip joints as well. Therefore, he was admitted our hospital for further examination in February 2011. He had a 7-year history of chronic hepatitis caused by hepatitis B virus infection, and had received lamivudine therapy for 2 years. Because the virus developed resistance to lamivudine, he received adefovir dipivoxil for 5 years before the development of the femoral neck fracture. After adefovir dipivoxil treatment, his liver function was restored. Radiography showed femoral neck fractures (right, Garden III fracture; left, Garden IV fracture) and a distal right tibial fracture (Figure 1a) [7]. Magnetic resonance imaging (MRI) of both hip joints showed fractures across the right and left femoral neck and bone edema, which had low intensity on T1-weighted images and high intensity on T2-weighted images (Figure 1b). ^99m^Tc-hydroxymethylene diphosphonate (HMDP) whole-body bone scintigraphy showed increased uptake of the radiotracer in the calvaria, maxilla, both scapulae, ribs, both femoral necks, right condyle of the femur, right tibia, and both tarsi (Figure 1c). He showed hypophosphatemia (2.0 mg/dL; normal range, 2.5–4.5 mg/dL) and increased levels of alkaline phosphatase (ALP, 1594 IU/L; normal range, 115–359 IU/L). Furthermore, he showed normal serum creatinine (0.7 mg/dL; normal range, 0.4–0.7 mg/dL), blood urea nitrogen (BUN, 12.3 mg/dL; normal range, 8.0–22.0 mg/dL), intact parathyroid hormone (PTH, 19 pg/mL; normal range, 10–65 pg/mL), and 1,25-dihydroxyvitamin D3 (40.0 pg/mL; normal range, 20–60 pg/mL) levels. Urinalysis revealed proteinuria. A 24-h study showed increased urinary excretion of phosphate (1004 mg/day; normal range, 70–220 mg/day), calcium (471.0 mg/day; normal range, 100–300 mg/day), *N*-acetylglucosaminidase (11.8 U/L; normal range, <7.0 U/L), and β2-microglobulin (64,579 μg/L; normal range, 230 μg/L). These findings indicated hypophosphatemia and hyperphosphaturia (increased levels of ALP). However, because the patient had normal levels of 1,25-dihydroxyvitamin D3, we considered that the impaired phosphate reabsorption could have been caused by dysfunction of the proximal renal tubule dysfunction and not by deficiency of vitamin D. Urinalysis and examination of urine samples collected over 24 h showed increased levels of *N*-acetylglucosaminidase and β2-microglobulin as well as phosphate wasting, which also indicated that these symptoms were caused by dysfunction of the proximal renal tubule.

**Figure 2:**
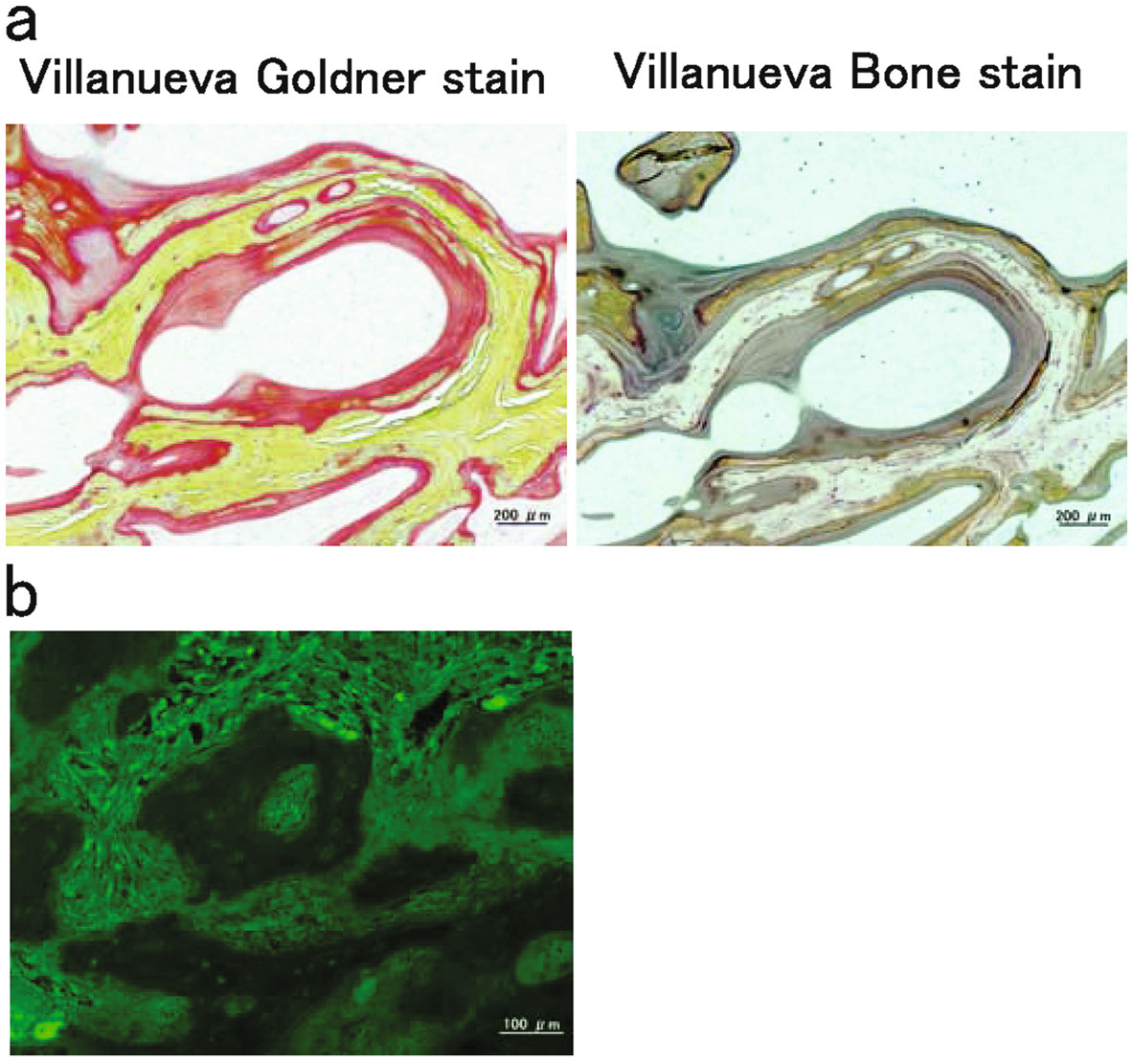
**Pathological examinations.a**: Mineralized bone tissues are colored in green and nonmineralized osteoid tissues are shown in orange by Villanueva-Goldner stain. Mineralized bone tissues are colored in purple and nonmineralized osteoid tissues are shown in the clear zone by Villanueva bone stain. The osteoids volume/mineralized bone volume ratio was 20.7% (normal range, less than 10%). Osteoid thickness was 25.1 μm (average, less than 12.5 μm). **b**: Tetracycline labeling examination showed no double-labeling pattern. These findings indicate mineralization deficiency and osteomalacia.

On the basis of these findings, we made a diagnosis of osteomalacia and pathologic fractures due to Fanconi’s syndrome secondary to adefovir therapy (10 mg/day). We conducted preoperative examinations to perform total hip arthroplasty. Prolonged bleeding time was observed by platelet aggregation failure and coagulation factor deficiency (Table 1). The coagulation disorder was suggested to have been caused by chronic hepatitis. The bleeding time was normalized by platelet transfusion. or double-labeling analysis, 1000 mg of tetracycline was orally administered at 10-day intervals. A 2-step procedure was performed under the same general anesthetic. During the first part of the procedure, biopsy of the iliac bone was performed, and during the second stage of the procedure, total hip arthroplasty was performed using a Zimmer implant (cemented collarless polished taper. stem, cementless trabecular metal modular acetabular cup, 36-mm head; Figure 1d). Because the acetabular roof bone was too fragile to support the acetabular components, bone fragment autografts prepared from the left femoral head were transplanted at the acetabular roof. The patient received intravenous antibiotics for 3 days. On the first postoperative day, the patient began rehabilitation under the supervision of a physiotherapist. He began using crutches for ambulation on postoperative day 7, with progressive weight-bearing as tolerated. The time to full weight-bearing was 3 weeks after the operation. The iliac bone and femoral head samples were fixed and stained using Villanueva bone stain and Villanueva–Goldner counterstain. The osteoid volume/mineralized bone volume ratio was 20.7% (average, <10%) and osteoid thickness was 25.1 μm (average, <12.5 μm; Figure 2a). Examination using tetracycline labeling showed no double-labeling pattern (Figure 2b) [8]. These findings confirmed that the pathological fractures were caused by osteomalacia (reviewed in [8]). After surgery, adefovir dipivoxil was switched with entecavir hydrate, and eldecalcitol and alendronate sodium hydrate were administered. These treatments normalized the blood phosphate level. The Japanese Orthopaedic Association Hip Score for the hip joints was 73 points at 2 months after surgery. He did not show any new pathological fractures.

**Table 1:** Title: coagulation factors

	**Activity**	**Normal range**
Coagulation factor II	46.6%	(66.0–118.0)
Coagulation factor III	65.1%	(73.0–122.0)
Coagulation factor VII	65.6%	(54.0–162.0)
Coagulation factor VIII	89.8%	(78.0–165.0)
Coagulation factor IX	43.5%	(67.0–152.0)
Coagulation factor X	54.3%	(58.0–200.0)
Coagulation factor XI	43.5%	(75.0–137.0)
Von Willebrand factor	200%	(50.0–150.0)

## Discussion

Adefovir dipivoxil is a commonly used antiviral agent in the treatment of chronic hepatitis B or HIV infection [9]. Fanconi’s syndrome has been recognized as a complication of high-dose adefovir dipivoxil therapy (dose, 60–120 mg/day) in the treatment of HIV infection [10]. Few studies have reported severe hypophosphatemia with 10 mg/day adefovir dipivoxil therapy [11-14]. In addition, to our knowledge, this is the first report of pathological femoral neck fracture associated with adefovir dipivoxil-induced osteomalacia treated by total hip arthroplasty. When orthopaedic surgeons encounter adefovir dipivoxil–treated chronic hepatitis B patients with pathological hip fractures, the patients’ renal function and levels of electrolytes, including calcium and phosphorus, should be carefully monitored.

Fanconi’s syndrome results from dysfunction of the proximal renal tubule, causing impaired reabsorption of amino acids, urate, bicarbonate, and phosphate and increased excretion of these solutes into the urine. The pathophysiology of proximal renal tubule dysfunction is thought to be an increase in the adefovir dipivoxil concentration in the mitochondria mediated by inhibition of several ATP-dependent transporters [15,16]. Patients with Fanconi’s syndrome show low phosphate levels (because of renal phosphate loss) and normal levels of calcium, 25-hydroxyvitamin D, 1,25-dihydroxyvitamin D, and PTH and increased ALP levels. Radiography and bone scan showed multiple patterns of osteomalacia. Our findings were consistent with those in previous reports [17].

Entecavir is more effective than adefovir dipivoxil, with a favorable safety profile and low incidence of resistance [18]. We switched adefovir dipivoxil with entecavir hydrate as previously reported [19,20]. Entecavir may be a good treatment choice. In addition to adefovir dipivoxil, the patient received oral administration of lamivudine, rebamipide, rabeprazole sodium, and methylprednisolone. These drugs may have caused Fanconi’s syndrome. After the patient’s condition was diagnosed as Fanconi’s syndrome, adefovir dipivoxil was replaced with entecavir hydrate. Thereafter, the symptoms of Fanconi’s syndrome improved. These findings suggested that adefovir dipivoxil caused Fanconi’s syndrome and osteomalacia.

In conclusion, orthopaedic surgeons should be aware of osteomalacia and pathologic fractures caused by adefovir dipivoxil administered as anti-hepatitis B virus therapy. In addition to the above-mentioned antiviral agent, ifosfamide, valproic acid, aminoglycosides, methyl-3-chromone, paraquat, l-lysine, calcineurin-inhibitor, or tetracycline may cause hypophosphatemic osteomalacia; therefore, serum ALP and phosphorus levels of patients receiving these drugs should be monitored [21-28].

## Consent

Written informed consent was obtained from the patient and his family for publication of this case report. A copy of the written consent is available for review by the Editor-in-Chief of the journal.

## Abbreviations

HIV: Human immunodeficiency virus; MRI: Magnetic resonance imaging; HMDP: ^99m^Tc-hydroxymethylene diphosphonate; ALP: Alkaline phosphatase; BUN: Blood urea nitrogen; PTH: Parathyroid hormone.

## Competing interests

The authors declare that they have no competing interests.

## Authors’ contributions

MT, YA, and SN were responsible for data collection. TS, YI, and SK, were responsible for literature search and manuscript preparation. MH, YS, and HK performed microscopic examinations, and JK performed surgery. All authors have read and approved the final manuscript.
